# Correlation between the Signal Intensity Alteration of Infrapatellar Fat Pad and Knee Osteoarthritis: A Retrospective, Cross-Sectional Study

**DOI:** 10.3390/jcm12041331

**Published:** 2023-02-07

**Authors:** Zheng Liu, Jiangyi Wu, Wei Xiang, Jinhui Wu, Shu Huang, Yizhao Zhou, Hui Xia, Zhenhong Ni, Baorong Liu

**Affiliations:** 1 Department of Joint Surgery and Sport Medicine, The First Affiliated Hospital of Hunan Normal University, Changsha 410000, China; 2 Department of Sports Medicine and Rehabilitation, Peking University Shenzhen Hospital, Shenzhen 518036, China; 3 State Key Laboratory of Trauma, Burns and Combined Injury, Department of Rehabilitation Medicine, Daping Hospital, Army Medical University, Chongqing 400042, China; 4 Surgery Department I, The First Affiliated Hospital of Hunan Normal University, Changsha 410000, China

**Keywords:** osteoarthritis, IPFP, signal intensity alteration, MRI

## Abstract

Infrapatellar fat pad (IPFP) inflammation is a common pathological manifestation in knee osteoarthritis (OA). However, the significance of IPFP signal intensity alteration for clinical diagnosis and treatment of knee OA needs further research. We assessed IPFP signal intensity alteration (0–3), IPFP maximum cross-sectional area (CSA) and IPFP depth, meniscus injury, bone marrow edema, and cartilage injury from magnetic-resonance imaging (MRI) in 41 non-KOA patients (K-L grade 0 and grade I) and 68 KOA patients (K-L grade 2,3 and 4). We found that IPFP signaling was altered in all patients with KOA whose alteration was closely related to the K-L grading. We found that the IPFP signal intensity was increased in most OA patients, especially the ones in the late stage. There were significant differences in IPFP maximum CSA and IPFP depth between groups in KOA and non-KOA patients. Moreover, Spearman correlation analysis showed that IPFP signal intensity was moderately positively correlated with age, meniscal injury, cartilage injury, and bone marrow edema, and negatively correlated with height, while not correlated with visual analogue scale (VAS) scoring and body mass index (BMI). In addition, women have higher IPFP inflammation scores on MRI than men. In conclusion, IPFP signal intensity alteration is associated with joint damage in knee OA, which may have clinical significance for diagnosing and treating KOA.

## 1. Introduction

Osteoarthritis (OA) is a kind of joint degenerative disease mainly manifested by a series of comprehensive symptoms, such as cartilage injury, subchondral bone changes, osteophyte formation, synovial inflammation, and so on. OA often causes pain and joint deformity, which may lead to limb disability [1]. However, due to its unclear etiology and various pathogenic factors, the clinical treatment of early osteoarthritis mainly involves analgesic, anti-inflammatory, physical therapy and other programs to relieve symptoms [2]. Surgical operations are often required at the terminal stage, but there is still no effective treatment to cure and reverse pathological changes [3,4,5,6]. Therefore, how to achieve the goal of early treatment through early diagnosis has become the common goal of clinicians and scientists.

The infrapatellar fat pad (IPFP) is a fatty tissue structure within the knee joint. The IPFP is located below the patella, and it is an intracapsular but extrasynovial structure [7]. Due to the special location of the IPFP and the major composition of its collagen matrix, it is believed that the IPFP has a buffering effect on shock absorption during knee joint activities and is able to promote the distribution of synovial fluid [8,9]. The IPFP fills the space between the femur, tibia, and patella to stabilize the patella, thereby protecting the knee from mechanical injury, and, in addition, the IPFP can also act as a shock absorbing pad between the infrapatellar tendon and the anterior tibial plateau [10]. Changes in the IPFP due to OA may affect the biomechanical properties of the IPFP by impairing its ability to absorb compressive forces and gravity on the knee joint, which ultimately aggravate joint damage [11]. It was reported that the IPFP interacts with knee cartilage, synovium, subchondral bone and other periarticular tissues in the process of osteoarthritis [1,12]. Eymard, F. et al. found that IPFP in knee OA patients was characterized by inflammatory infiltration and vascularization, and increased interlobular septal thickness compared with healthy patients [13]. A 5-year follow-up study found that the pathological manifestations of IPFP were associated with OA symptoms and advanced osteoarthritis processes [14]. A longitudinal study showed that IPFP had a protective effect against knee pain and cartilage damage in older women with osteoarthritis. [15]. One randomized control trial found that removal of the IPFP during TKA may increase the proportion of patients with postoperative anterior knee pain, suggesting that the IPFP should be preserved as much as possible during TKA, provided that the surgical field is fully exposed during surgery [16].

Current studies have found that changes in the signal intensity of the IPFP under magnetic resonance imaging (MRI) could be observed in the early stages of KOA, which is similar to the changes in bone marrow damage and cartilage defects [17,18]. The maximum cross-sectional area (CSA) and IPFP depth are usually used to describe the size of the IPFP in MRI. Cowan, S.M. et al. reported that patients with patellofemoral joint osteoarthritis had larger IPFP volumes than asymptomatic patients, and that larger IPFP volumes were often accompanied by worse pain [19]. However, some studies have found that the maximum CSA of the IPFP is negatively correlated with radiological OA and knee pain, suggesting that the IPFP may play a protective role in OA progression [20,21]. Similarly, Ragab, E. et al. reported a significant negative correlation between IPFP area and OA grade, as well as MRI manifestations of OA [22]. These suggested that the volume of IPFP is related to a variety of factors, including OA grade, subtype, and genetic background. Ballegaard C.D. et al. found that perfusion variables in contrast-enhanced MRI (CE-MRI) could assess the degree of IPFP inflammation, and found that severe inflammation of IPFP was associated with severe pain in patients with KOA [23]. In brief, IPFP signal intensity alternation could be radiographically assessed and graded with MRI. Moreover, this change may be strongly related to disease progression in KOA. However, in cross-sectional studies, the correlation between IPFP signal intensity changes and osteoarthritis features in non-KOA and KOA patients still needs to be further studied.

In this study, we selected 41 non-KOA and 68 KOA patients, compared the changes of IPFP signal intensity between the two groups through baseline data (such as BMI, VAS score, age, gender, side, etc.), X-ray images, and MRI imaging changes, and analyzed the correlation between the IPFP and KOA features. We hope that this cross-sectional study can further improve the level of diagnosis and intervention for KOA based on the IPFP.

## 2. Materials and Methods

### 2.1. Study Design and Participants

This study was a retrospective, longitudinal cohort study. The research protocol conducted in this study was reviewed and approved by the Institutional Review Board of the Hunan Provincial People’s Hospital Clinical Center, and informed consent was obtained from all study participants.

From January 2022 to October 2022, 212 patients who underwent knee X-ray and MRI were selected. The inclusion criteria of the KOA group were as follows: patients whose K-L grading was II, III, IV were diagnosed as osteoarthritis were included in the study group according to K-L grading [24], received relevant imaging examination, and agreed to sign the informed consent form; age ≥ 40. The inclusion criteria of the non-KOA group were as follows: patients whose K-L grading was 0 and I were diagnosed as non-KOA and were included in control group; they received relevant imaging examination and agreed to sign the informed consent form; age ≥ 40. The exclusion criteria of the two groups were as follows: diagnosis of ankylosing spondylitis, rheumatoid arthritis, psoriatic arthritis, or any other type of chronic immune disease; history of knee injury or surgery; systemic corticosteroid treatment, intra-articular injection or other drugs for bone-related diseases; congenital varus; contraindications of magnetic resonance examination, and rejection of participants.

**Figure 1:**
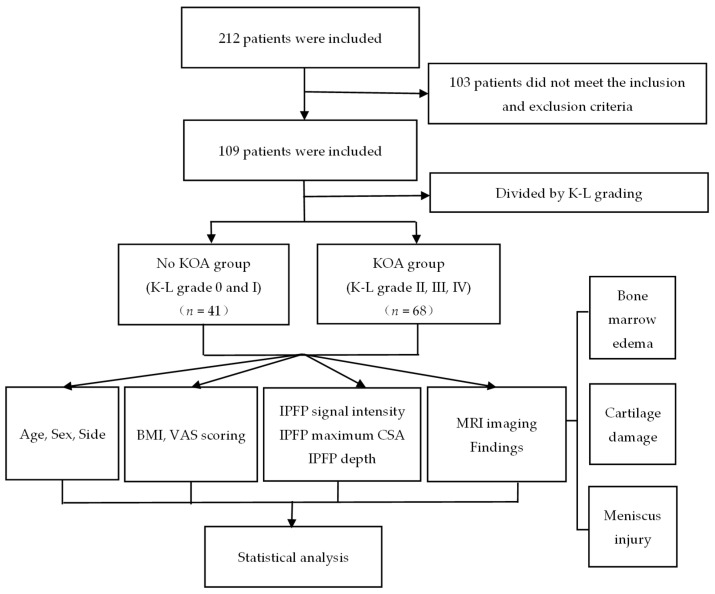
Patient’s flow chart. Patient profiles and the groups included in the study. K-L, Kellgren-Lawrence; KOA, knee osteoarthritis; BMI, body mass index; VAS, visual analogue scale; CSA, cross-sectional area; MRI, magnetic-resonance imaging.

Finally,109 patients were included according to the inclusion and exclusion criteria. According to the K-L classification on X-ray films, the patients were divided into non-KOA group (K-L grade 0 and grade I) and KOA group (K-L grade II and above). The IPFP signal intensity alternation was scored on MRI (Figure 1).

**Figure 2:**
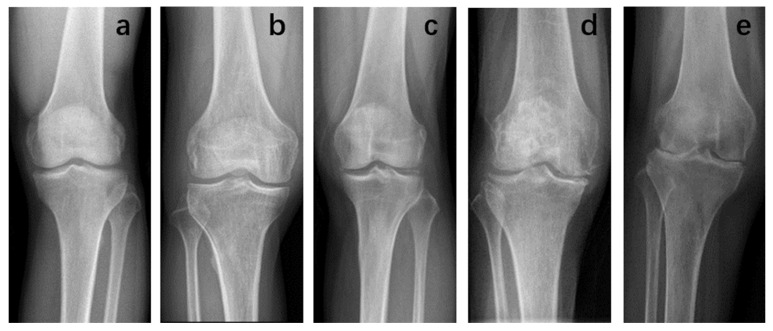
K-L classification. The patient was graded on an X-ray of the knee joint according to the method previously described. (**a**) Grade 0; (**b**) Grade I; (**c**) Grade II; (**d**) Grade III; (**e**) Grade IV.

According to the K-L classification of knee X-ray evaluation: grade 0, no osteophytes and narrowing of the joint space; grade I represents no obvious narrowing of the joint space, possible osteophytes; grade II, mild osteophytes, normal or suspicious narrowing of the joint space; grade III, moderate Osteophytes with stenotic subchondral sclerosis of the joint; and grade IV represents obvious osteophytes, with markedly narrowed joint septa and severe sclerosis of the subchondral bone (Figure 2).

### 2.2. MRI Acquisition

MR Imaging: 3.0T MR Scanner (Philippe Achieva, Amsterdam, Netherlands) was used for MR Imaging of the knee joint, with a 16-channel special coil for the knee joint, scanning sequence parameters: ➀ Sagittal PDW-SPAIR: repetition time (TR) 3693 ms, echo time (TE) 30 ms, flip angle (FA) 90°, field of view (FOV) 180 mm × 180 mm, moment array 360 × 270, slice thickness 3.5 mm, slice spacing 0.35 mm, slice number 24, acquisition frequency 1, scan time 155 s. ➁ Coronal PDW-SPAIR: TR4565 ms, TE30 ms, FA 90°, FOV180 mm × 160 mm, moment array 360 × 249, slice thickness 3.5 mm, slice spacing 0.35 mm, slice number 24, acquisition times 2, scan time 146 s. ➂ Sagittal T2WITSE: TR 3204 ms, TE 80 ms, FOV 180 mm × 180 mm, moment array 328 × 251, slice thickness 3.5 mm, interslice distance 0.35 mm, acquisition time 2, acquisition time 82 s; ➃ Coronal T1WITSE: TR 568 ms, TE 20 ms, FA 90°, FOA 180 mm × 160 mm, slice thickness 3.5 mm, slice spacing 0.35 mm, slice number 24 layers, acquisition frequency 1, scan time 92 s. All imaging analyses were performed on the software MIMICS 16.0 (Materialism, Leuven, Belgium). The acquired images were separately analyzed in a blinded fashion by two trained senior radiologists, and one of them reanalyzed all patients 4 weeks later.

### 2.3. Outcome Measures

**Figure 3:**
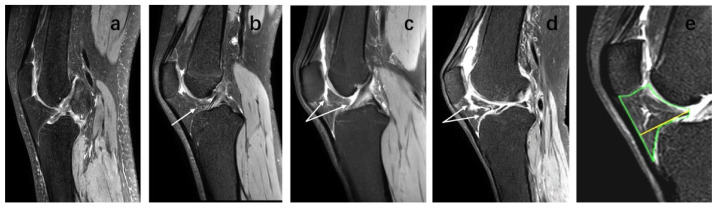
IPFP signal intensity alteration, IPFP maximum cross-sectional area (CSA) and IPFP depth on sagittal planes of T2-weighted images. IPFP signal intensity alteration graded as follows: (**a**) Grade 0 = none; (**b**) Grade 1 ≤ 10% of the area; (**c**) Grade 2 = 10–20% of the area; (**d**) Grade 3 ≥ 20% of the area. The area indicated by the white arrow within the IPFP represent the high signal regions. (**e**) The green area indicates the IPFP maximum CSA. The yellow line which perpendicular to the patellar tendon was drawn from the anterior to posterior surface indicates IPFP depth.

On sagittal T2-weighted MR Images, the IPFP boundary was manually delineated to measure the IPFP, and the maximum cross-sectional area (CSA) was calculated. The maximum sagittal thickness of the IPFP was measured manually, and a line perpendicular to the patellar tendon was drawn from the anterior to posterior surface, and this was used to indicate the IPFP depth. IPFP signal intensity alteration on MRI were assessed by an experienced orthopedic surgeon and an experienced radiologist using T2-weighted MRI. The sagittal plane with the most significant increase in IPFP signal was selected as the plane of interest for study, and edema was defined as high signal on the fat-suppression sequence. The sagittal plane of the IPFP was segmented using MIMICS, and the area indicated by the white arrow within the IPFP represented the high signal regions [25] (Figure 3). IPFP signal intensity alteration was graded as follows: Grade 0 = none; Grade 1 ≤ 10% of the area; Grade 2 = 10–20% of the area; Grade 3 ≥ 20% of the area [18]. Two observers observed alone, and a random cross-check was performed with the other observer. The intra-class correlation coefficient (ICC) was 0.94 for intra-observer reliability, and inter-observer reliability was 0.93.

### 2.4. Statistical Analysis

Statistical analysis was performed using IBM SPSS Statistics 25.0 (IBM, Armonk, NY, USA). Data were reported as mean ± standard deviation (SD) for continuous parameters and as frequencies and percentages for categorial parameters. Descriptive statistics were performed on all parameters, and the data were expressed as means with 95% confidence intervals (CI). The Chi-square test was used for the evaluation of categorical variables, and the *t*-test or Mann-Whitney test was used for the evaluation of continuous variables. The Spearman correlation test was used to analyze the correlation between knee osteoarthritis features (including BMI value, age, gender, location, knee imaging changes, etc.) and maximum CSA of IPFP and IPFP signal intensity alternation on MRI. The correlation coefficient (r) = 0.2~0.4 is mild correlation, 0.4~0.6 is moderate correlation, 0.6~0.8 is strong correlation, and r > 0.8 is extremely strong correlation. A difference of *p* < 0.05 was considered as statistically significant.

## 3. Results

### 3.1. Patients

A total of 109 knees were included and classified as non-KOA group (41 knees) and KOA group (68 knees). The gender, weight, BMI, left and right sides and other basic information are shown in Table 1.

### 3.2. The Correlation between IPFP Signal Intensity Alternation, IPFP Maximum CSA, IPFP Depth, K-L Grading and OA Features

The IPFP signal intensity alternation was measured using the previously described method for groups. We counted 41 cases in group A, including 24 cases in grade 0 and 17 cases in grade 1. There were 68 cases in group B, including 25 cases in grade 2, 25 cases in grade 3, and 18 cases in grade 4.

According to Spearman correlation analysis, IPFP signal intensity under MRI showed a moderate positive correlation with age (r = 0.499, *p* < 0.01), and a weak negative correlation with height (r = −0.288, *p* = 0.002); showed a very weak negative correlation with gender (r = −0.196, *p* = 0.041); there was no significant correlation with side (r = 0.017, *p* = 0.86), body weight (r = −0.088, *p* = 0.365),BMI (r = 0.151, *p* = 0.117) and VAS (r = 0.108, *p* = 0.262) (Table 2). Interestingly, IPFP signal intensity alternation had a weak negative correlation with height, but not a significant correlation with weight and BMI, suggesting that increased height may be a protective factor for IPFP signal intensity alternation, but larger sample sizes are needed to confirm this hypothesis.

The IPFP maximum CSA showed a weak negative correlation with age (r = −0.269, *p* = 0.005) and a very weak positive correlation with gender (r = 0.193, *p* = 0.044) (Table 2). In addition, IPFP maximum CSA was not significantly correlated with BMI, VAS score, height, weight, and side.

The IPFP depth showed a weak negative correlation with age (r = −0.335, *p* < 0.01) and VAS scoring (r = −0.227, *p* = 0.018) (Table 2). Additionally, IPFP depth was not significantly correlated with other features.

The K-L grading was strongly positively correlated with age (r = 0.754, *p* < 0.01), and weakly negatively correlated with gender (r = −0.365, *p* < 0.01) and body height (r = −0.341, *p* < 0.01). In addition, there was a weak positive correlation with BMI (r = 0.318, *p* < 0.01) (Table 2). These findings also coincide with previous studies suggesting that women, advanced age, and high BMI are risk factors for knee osteoarthritis.

### 3.3. The Correlation between IPFP Signal Intensity Alternation, IPFP Maximum CSA, IPFP Depth, K-L Grading and Radiographic Changes

We found that IPFP signal intensity under MRI was moderately positively correlated with meniscus injury (r = 0.532, *p* < 0.01), cartilage injury (r = 0.519, *p* < 0.01) and bone marrow edema (r = 0.482, *p* < 0.01) (Table 3).

The IPFP maximum CSA was weakly negatively correlated with meniscus injury (r = −0.206, *p* < 0.05) and cartilage injury (r = −0.340, *p* < 0.01) (Table 3).

The IPFP depth was weakly negatively correlated with meniscus injury (r = −0.252, *p* < 0.05) and cartilage injury (r = −0.359, *p* < 0.01) (Table 3).

In addition, The K-L grading was strongly positively correlated with meniscus injury (r = 0.610, *p* < 0.01) and cartilage injury (r = 0.728, *p* < 0.01), and moderate positively correlated with bone marrow edema (r = 0.576, *p* < 0.01) (Table 3).

### 3.4. The Differences of Parameters between KOA Group and Non-KOA Group

There were statistically significant differences between the KOA and non-KOA groups in meniscus injury, bone marrow edema, cartilage injury, IPFP score, IPFP depth and IPFP maximum CSA, while no significant differences were found in VAS score (Table 4).

## 4. Discussion

In this study, we found that the IPFP signal intensity was increased in most OA patients, especially those in the late stage. There were significant differences of IPFP maximum cross-sectional area (CSA) and IPFP depth between groups in patients with KOA and non-KOA patients. Moreover, Spearman correlation analysis showed that IPFP signal intensity was moderately positively correlated with age, meniscal injury, cartilage injury, and bone marrow edema, and negatively correlated with height, while not correlated with visual analogue scale (VAS) scoring and body mass index (BMI). In addition, women have higher IPFP inflammation scores on MRI than men.

Our and previous studies have found that IPFP signal intensity alternation under MRI is a very effective imaging method for evaluating OA, indicating that IPFP is closely related to the disease progression and pathological changes in OA. These findings suggest that IPFP signal intensity alterations may have clinical significance for diagnosis and intervention of OA.

The K-L classification is the most widely used clinical imaging method for osteoarthritis. It scans the knee joints of patients with X-rays and evaluates the formation of osteophytes, joint space, cartilage and subchondral bone. OA is divided into grades I-IV [26]. Previously, some researchers believed that IPFP signal intensity on MRI could predict the occurrence of radiological OA (ROA) [27]. This pattern was also found in our study: an increase in IPFP inflammation score was always accompanied by a higher K-L grade of osteoarthritis; that is, there was a significant correlation between IPFP inflammation score and K-L grade under MRI.

In the cases we collected, we found some interesting phenomena. There were some cases without OA but with IPFP signal change. All advanced OA had moderate or greater IPFP signal intensity changes (IPFP scoring ≥ 2), while this proportion was also nearly half in non-advanced OA. This suggests that the late stage of knee OA is often accompanied by changes in IPFP signal intensity. This is also consistent with a previous report [10]. However, we have not found the representative characteristics of these cases based on present data. This is really important for IPFP research, and we will pay more attention to this issue in the future.

With more in-depth understanding of osteoarthritis, its complex etiology and the interaction of internal and external structures in joints have become the focus of scientists’ attention. As the largest and most complex joint in the human body, the knee joint, articular cartilage, subchondral bone, synovium, infrapatellar fat pad, ligaments, and skeletal muscles jointly promote or inhibit the progression of osteoarthritis [28,29,30,31,32,33,34].

Cartilage damage, bone marrow edema, and meniscus degeneration or damage are common clinicopathological changes in patients with osteoarthritis, especially in end-stage osteoarthritis [35]. Trauma can lead to damage to the bone or cartilage, making the joint more vulnerable to further damage, while damage to the ligaments or meniscus can adversely affect the biomechanics of the joint. Cartilage, subchondral bone, and synovium may all play key roles in disease pathogenesis and may also be involved in systemic inflammation [35,36].

MRI can observe the changes in IPFP signal intensity in patients with knee OA, thus providing direct evidence for the involvement of IPFP in the pathogenesis of knee osteoarthritis. At the same time, MRI has many advantages over X-rays, and can use three-dimensional imaging and high resolution to evaluate joint structure. Therefore, it is more sensitive in detecting early structural changes [37]. Our study suggested that IPFP inflammation score under MRI was positively correlated with meniscus injury, cartilage injury, bone marrow edema, and the correlation was significant. It should be emphasized that existing evidence could not strongly support that IPFP signal changes occur earlier than OA progression on X-ray. Davis JE et al. conducted a 2-year follow-up study and found that patients with IPFP signal intensity changes without radiographic OA changes may be at high risk for OA acceleration, which may be characterized primarily by local inflammation [38]. In our study, we found that IPFP signaling changes may be a potential risk factor for OA. In the future, we need to increase the number of participants and conduct long-term follow-up studies to find IPFP signal features of early OA in MRI imaging, so as to help early diagnosis of OA.

The maximum cross-sectional area (CSA) of IPFP and the depth of IPFP are commonly used to describe the size of IPFP in MRI [39]. In our study, compared with the non-OA group, the OA group had smaller IPFP maximum CSA and IPFP depth. Similarly, Fontanella, CG. et al. reported that IFP volume, depth, length of femoral and tibial arch in patients with moderate and end-stage osteoarthritis were decreased compared with the control group, suggesting that IPFP is a protective factor for OA [40]. However, another study has reported larger IPFP volumes in patients with patellofemoral osteoarthritis than in asymptomatic patients, and larger IPFP volumes tend to be associated with more severe pain [19]. The volume of IPFP may be related to a variety of factors, including OA grade, subtype, and genetic background. Therefore, more in-depth research is needed to clarify the characteristics of IPFP volume in OA progression.

In addition, there are many risk factors for osteoarthritis. Wang et al. believe that obesity is one of the most influential and variable risk factors [41]. A. W, Ferrante Jr. and Chang, J. believe that obesity is related to degenerative and inflammatory responses, and adipose tissue may secrete molecules linking obesity and osteoarthritis [42,43]. However, in our results, BMI was not significantly correlated with IPFP signal intensity alternation, while there was a weak positive correlation with K-L grading. This suggests that BMI may be somewhat correlated with IPFP, but it is not reflected on MRI imaging. Unexpectedly, we found that height was negatively correlated with IPFP signal changes and OA radiological grade. De Jong, AJ. et al. reported that greater IPFP volume was associated with greater height [44]. Furthermore, some studies have found a possible relationship between the genetic basis of height and OA, possibly mediated through altered bone growth and development [45]. The above studies indicated that this may partially explain the relationship between height and OA. More in-depth studies are needed in the future.

Women are often seen as a high-risk factor for osteoarthritis. The incidence of osteoarthritis of the knee, hip, and hand is greater in women than in men, and the incidence increases significantly in women before and after menopause [46]. Most researchers believe that this is related to the level of estrogen in women, although some studies have suggested that estrogen has a protective role in osteoarthritis, and it has also been linked to the pathogenesis of osteoarthritis [47,48]. This suggests that gender differences between men and women are not only explained by estrogen, but may also be related to other factors, such as muscle strength, lifestyle habits, and bone density. Our data analysis also suggested that the IPFP inflammation score was negatively correlated with gender; that is, the incidence of women was higher.

Advanced age is regarded as a separate risk factor for osteoarthritis [49]. At first, it was found that senescent chondrocytes increased in osteoarthritis patients. With the deepening of research, researchers found that in addition to chondrocytes, senescent synovial fibroblasts were involved in the progression of osteoarthritis [50,51]. Recent studies suggest that the inflammatory environment induced by aging-associated secreted phenotype (SASP) factors is involved in cartilage degeneration and subchondral bone remodeling, ultimately leading to cartilage loss and the progression of osteoarthritis [52]. In our study, we also found a moderate positive correlation between the signal intensity alteration of IPFP and age, suggesting that IPFP is closely related to aging.

Pain is the most common clinical manifestation in patients with osteoarthritis. Some studies have found that the change in IPFP signal intensity is positively correlated with the increase in knee joint pain [15]. However, in our study, there was no significant correlation between VAS score and IPFP signal intensity change. At the same time, some scholars believe that there is no obvious correlation between the maximum area and signal intensity of IPFP with knee joint pain, suggesting that in the process of osteoarthritis pain, IPFP may affect pain through biochemical changes that do not affect the morphology and signal intensity of IPFP under MRI, such as stimulating the biochemical interaction of nociceptive fibers to drive pain [53,54].

However, the following issues still need to be solved in the future: the relationship between serum inflammatory factor levels and MRI manifestations of IPFP; the correlation between MRI findings and pathological changes of IPFP; and changes in MRI features and OA symptoms after clinical interventions such as rehabilitation training.

This study has several limitations that should be acknowledged. Firstly, the patients with KOA are older than non-KOA patients in our study; we will add the number of participants so as to avoid this limitation in the future. Secondly, although we found increased IPFP signal intensity in the majority of OA patients, especially in those with advanced OA, there is insufficient evidence to suggest that changes in IPFP signal precede radiographic OA progression, and future studies with more participants and long-term follow-up from the baseline period may be needed. Thirdly, only qualitative analysis of cartilage damage and bone marrow edema was performed in this study, which may have resulted in some error, therefore further quantitative or semi-quantitative analysis can be performed in the future. Due to the limitation of the condition, pathological examination of synovial inflammation was not performed, and there may be some synovitis caused by non-osteoarthritis.

## 5. Conclusions

In brief, the present study demonstrates that the IPFP signal intensity alteration is associated with joint damage in knee OA, which may have clinical significance for diagnosing and treating KOA. Our data may provide new insight into the development of methods for the diagnosis and treatment of OA.

## Figures and Tables

**Table 1:** Patient Characteristics Between the non-KOA ^a^ and KOA Groups.

	No KOA	KOA	Z ^b^/χ^2^	*p*
Sex, n (male/female)	26/15	51/17	1.66	0.198
Age, year	46.76 ± 8.43	65.28 ± 9.03	−10.63	<0.01
Height, cm	166.68 ± 8.53	160.22 ± 7.23	4.22	<0.01
Weight, kg	63.40 ± 11.14	62.19 ± 8.13	0.66	0.513
BMI ^c^, kg/m^2^	22.78 ± 3.44	24.27 ± 3.13	−2.31	0.023
Side, n (left/right)	21/20	29/39	0.76	0.384

^a^. KOA: Knee Osteoarthritis; ^b^. Z: Z value, statistic of the Mann-Whitney test; ^c^. BMI: Body Mass Index.

**Table 2:** Correlation of gender, height, weight, age and location, VAS ^a^ scoring and BMI with IPFP ^b^ signal intensity, IPFP maximum CSA ^c^, IPFP depth and K-L grading ^d^.

	IPFP Signal Intensity		IPFP ^b^ Maximum CSA ^c^		IPFP Depth		K-L Grading ^d^	
	r	*p*	r	*p*	r	*p*	r	*p*
Gender	−0.196	0.041	0.193	0.044	0.176	0.067	−0.365	<0.01
Height, cm	−0.288	0.002	0.153	0.112	0.093	0.337	−0.341	<0.01
Weight, kg	−0.088	0.365	0.021	0.829	−0.035	0.716	0.017	0.860
Age, year	0.499	<0.01	−0.269	0.005	−0.335	<0.01	0.754	<0.01
Side	0.017	0.86	0.038	0.697	−0.019	0.842	0.084	0.387
VAS ^a^	0.108	0.262	−0.133	0.169	−0.227	0.018	0.115	0.234
BMI, kg/m^2^	0.151	0.117	−0.112	0.248	−0.143	0.137	0.318	<0.01

^a^. VAS: Visual Analogue Score; ^b^. IPFP: infrapatellar fat pad; ^c^. CSA: cross-sectional area; ^d^. K-L grading: Kellgren-Lawrence grading.

**Table 3:** Correlation between imaging changes of the knee under MRI with IPFP signal intensity alternation, IPFP maximum CSA, IPFP depth and K-L grading.

	IPFP Signal Intensity		IPFP Maximum CSA		IPFP Depth		K-L Grading	
	r	*p*	r	*p*	r	*p*	r	*p*
Meniscus damage	0.532	<0.01	−0.206	0.031	−0.252	0.008	0.610	<0.01
Bone marrow edema	0.482	<0.01	−0.140	0.147	−0.175	0.069	0.576	<0.01
Cartilage damage	0.519	<0.01	−0.340	<0.01	−0.359	<0.01	0.728	<0.01

**Table 4:** The differences of parameters between KOA group and non-KOA group.

	No KOA	KOA	Z/χ^2^	*p*
Meniscus damage	30/11	14/54	28.379	<0.01
Bone marrow edema	27/14	7/61	36.787	<0.01
Cartilage damage	39/2	16/52	52.444	<0.01
IPFP scoring	N/A	N/A	39.656	<0.01
IPFP max CSA, mm^2^	706.83 ± 111.08	599.83 ± 137.26	4.224	<0.01
IPFP depth, mm	30.90 ± 3.52	28.28 ± 1.61	4.49	<0.01
VAS	2.44 ± 0.98	2.57 ± 1.26	−0.623	0.535

## Data Availability

The data that support this study are available from the author, Zheng Liu, upon reasonable request.
